# Role of nab-paclitaxel in metastatic breast cancer: a meta-analysis of randomized clinical trials

**DOI:** 10.18632/oncotarget.18900

**Published:** 2017-06-30

**Authors:** Yun Liu, Guoxin Ye, Dali Yan, Lei Zhang, Fan Fan, Jifeng Feng

**Affiliations:** ^1^ Department of Medical Oncology, Jiangsu Cancer Hospital, Jiangsu Institute of Cancer Research, Nanjing Medical University Affiliated Cancer Hospital, Nanjing 210009, China; ^2^ Division of Nephrology, Huashan Hospital, Shanghai Medical College, Fudan University, Shanghai 200040, China; ^3^ Department of General Surgery, Jiangsu Cancer Hospital, Jiangsu Institute of Cancer Research, Nanjing Medical University Affiliated Cancer Hospital, Nanjing 210009, China; ^4^ Department of Medical Oncology, No.2 Affiliated Hospital of Xuzhou Medical University, Xuzhou 221000, China

**Keywords:** nab-paclitaxel, taxanes, chemotherapy, breast cancer, meta-analysis

## Abstract

Whether nab-paclitaxel and conventional taxanes are equally effective for metastatic breast cancer (MBC) remains unclear. We conducted meta-analysis of trials that compared nab-paclitaxel-based chemotherapy with solvent-based paclitaxel (sb-paclitaxel) and docetaxel-based chemotherapy. A literature search was performed to identify articles that compared nab-paclitaxel-based chemotherapy with sb-paclitaxel or docetaxel-based chemotherapy for MBC. Four randomized controlled trials (1,506 patients) were identified from 1,268 reports. We detected equivalent overall response, overall survival, and survival probability (one-year, two-year). Grade 3 to 4 hematological and non-hematological toxicities were also comparable except that sensory neuropathy was more prominent for nab-paclitaxel-based chemotherapy (16.9% vs. 10%, odds ratio = 1.89, 95% confidence interval = 1.36–2.61, *P* < 0.001). No significant publication bias was detected. Consistent results stratified by treatment arm, study phase, treatment line, and study location were observed, except that overall response rate to nab-paclitaxel-based chemotherapy was significantly higher in the subgroup of randomized phase II trials, non-first-line treatment, and East Asian population. This meta-analysis failed to demonstrate advantages of nab-paclitaxel compared with sb-paclitaxel and docetaxel in patients with MBC. The newer agent was associated with increased sensory neuropathy, equivalent survival, and possibly increased overall response for some specific patients.

## INTRODUCTION

Breast cancer is the most common form of invasive cancer in women in developed and less developed countries, accounting for more than 1,000,000 new cases and 521,900 deaths occurring worldwide annually [1]. Despite a marked increase in choice of active agents, drugs result in modest influence on overall survival (OS). Currently, first-line therapy for metastatic breast cancer (MBC) include available taxanes, solvent-based paclitaxel (sb-paclitaxel), and docetaxel (Taxotere; Aventis Pharmaceuticals Inc., Bridgewater, NJ); they are also often used as adjuvant chemotherapy for patients with early-stage disease [2–5]. As they are hydrophobic, taxanes require solvents to enable parenteral administration. Sb-paclitaxel contains a combination of polyethylated castor oil and ethanol (Cremophor; Bristol-Myers Squibb, NJ, USA) as excipient, whereas docetaxel comprises polysorbate 80 and ethanol diluents.

Although sb-paclitaxel and docetaxel proved to perform significant actions against breast cancer and other solid tumors, emerging data indicate that polyethylated castor oil and polysorbate 80 directly contribute to severe toxicities observed in patients; these effects include hypersensitivity reactions, neutropenia, and sensory neuropathy [6, 7]. Additionally, active agents of these drugs may be trapped in solvent micelles, limiting their availability to tumors and prolonging systemic exposure, which in turn increases drug toxicity [8]. Sb-paclitaxel and docetaxel require premedication and special infusion sets and feature substantial “chair time” for drug administration. In case of sb-paclitaxel, the recommended infusion time spans 2–4 h [9]. Overall, these characteristics translate to increased costs for drug delivery and excessive burden to patients [10, 11].

Nab-paclitaxel (Abraxane; Abraxis BioScience, Los Angeles, CA), an albumin-bound 130nm particle form of paclitaxel, was developed to avoid toxicities associated with Cremophor vehicle in sb-paclitaxel [12–15]. Preclinical studies in animals demonstrated increased antitumor activity of nab-paclitaxel compared with equitoxic doses of sb-paclitaxel [16]. Previous clinical trials suggested that nab-paclitaxel may exhibit more significant efficacy and favorable safety profile compared with conventional taxanes in treatment of MBC [13, 17–19]. However, these findings were not consistent with those of a recent clinical trial [20], which demonstrated inferiority and toxicity of nab-paclitaxel compared with standard paclitaxel.

To elucidate the role of nab-paclitaxel in treatment of MBC, we conducted meta-analysis of randomized clinical trials to evaluate efficacy and toxicity of this drug compared with sb-paclitaxel and docetaxel. Results of our study will help facilitate therapeutic decision-making and optimize patient outcomes.

## RESULTS

### Characteristics of eligible studies

**Figure 1:**
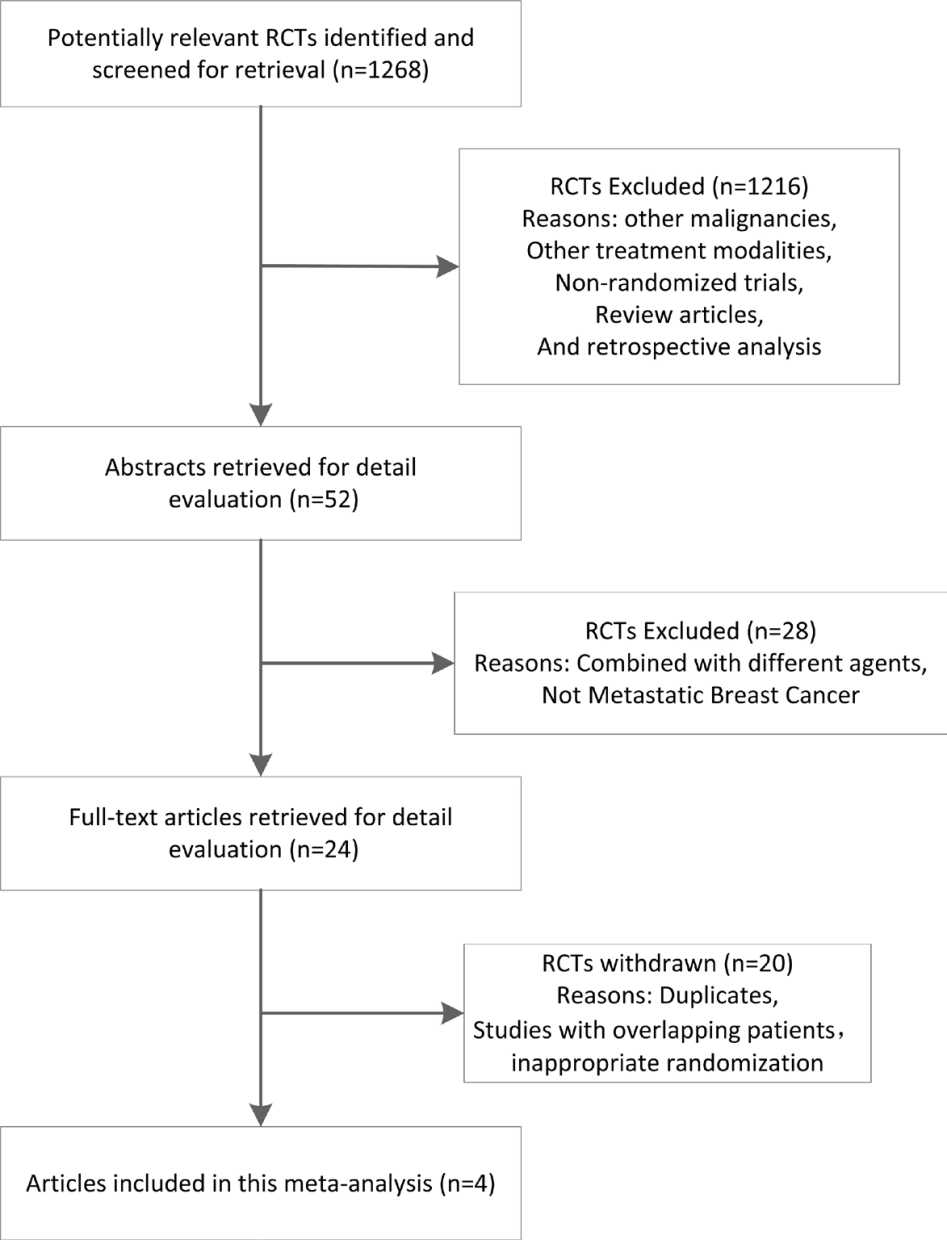
Stepwise procedures for searching databases and selecting eligible studies RCT, randomized controlled trials.

Figure 1 shows detailed steps of the search for eligible studies. After selection, four trials were identified [13, 17, 19, 20], and their data were obtained (Table 1). Analysis was conducted on individual data of 1,506 MBC (stage IIIB–IV) patients who were enrolled in trials and randomly assigned to receive chemotherapy with nab-paclitaxel (826 patients), sb-paclitaxel (606 patients), and docetaxel (74 patients). Patient characteristics were well-balanced between treatment regimens. Among four trials, two were randomized phase III trials [13, 20], and the remaining were randomized phase II trials [17, 19]. None of the studies was placebo-controlled or double-blind trials. Two trials did not employ complete first-line treatment. Review of original references clarified that these two trials excluded patients when they received adjuvant chemotherapy with taxanes (sb-paclitaxel or docetaxel) 12 months prior to study enrolment. However, detailed data were not provided. One trial compared nab-paclitaxel and sb-paclitaxel when they were used in combination with bevacizumab [20]. The trial also included other treatment arms in addition to the two arms considered for meta-analysis.

**Table 1:** Characteristics of the four trials comparing nab-paclitaxel-based with sb-paclitaxel and docetaxel-based chemotherapy

Study, year	Treatment line	Regimen	No. for analysis	Age, y	PS 0–1, *n* (%)	CR+PR (%)	Study phase	Jadad score	Study location
Gradishar et al./2005 [13]	1st line: 42% vs. 40% (NP vs. SP)	NP 260 mg/m^2^d1, q3w	229	53.1	215 (94)	76 (33)	III	3	Russia/Ukraine, US/Canada and UK
		SP 175 mg/m^2^d1, q3w	225	53.3	220 (98)	42 (19)			
Guan et al./2009 [17]	1st line: 59% vs. 60% (NP vs. SP)	NP 260 mg/m^2^d1, q3w	104	50	104 (100)	56 (54)	II	3	China
		SP 175 mg/m^2^d1, q3w	106	48.8	106 (100)	31 (29)			
Gradishar et al./2012 [19]	1st line	NP 300 mg/m^2^d1, q3w	76	51.7	69 (91)	28 (37)	IIb	3	Russia and US
		NP 100 mg/m^2^d1, 8, 15, q4w	76	55.4	72 (95)	34 (45)			
		NP 150 mg/m^2^d1, 8, 15, q4w	74	53.3	69 (93)	36 (49)			
		Doc 100 mg/m^2^d1, q3w	74	55.4	72 (97)	26 (35)			
Rugo et al./2015 [20]	1st line	NP 150 mg/m^2^d1, 8, 15, q4w + Bev 10 mg/kgd1, 15, q4w	267	54.3	267 (100)	91 (34)	III	3	US
		SP 90 mg/m^2^d1, 8, 15, q4w + Bev 10 mg/kgd1, 15, q4w	275	55.1	275 (100)	105 (38)			

Abbreviations: NP, nab-paclitaxel; SP, sb-paclitaxel; Doc, docetaxel; Bev, bevacizumab; PS, performance status; CR, complete response; PR, partial response.

We assessed quality of trials using three-question instrument reported by Jadad et al. [21]. All trials made statements of randomization and withdrawals, whereas none was described as double-blinded. Therefore, we assigned two points for all trials and judged that study quality was not a source of heterogeneity. Table 1 lists quality scores of trials.

### Overall response

**Figure 2:**
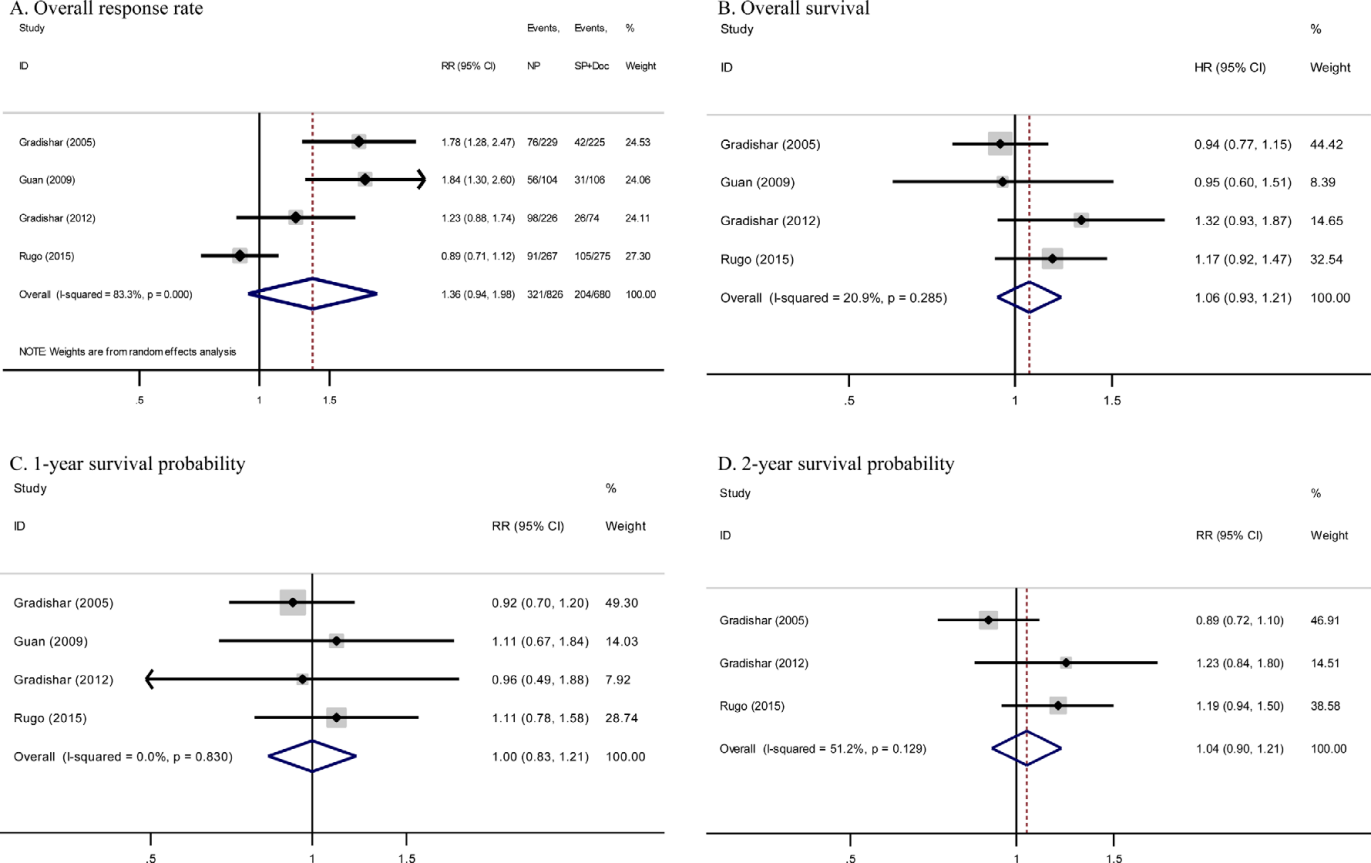
Forest plots estimating primary outcomes in comparison of nab-paclitaxel-based versus sb-paclitaxel and docetaxel-based chemotherapy (**A**) ORR, (**B**) OS, (**C**) one-year survival probability, and (**D**) two-year survival probability. NP, nab-paclitaxel; SP, sb-paclitaxel; Doc, docetaxel; CI, confidence interval; HR, hazard ratio; RR, risk ratio.

Overall response case numbers were presented in all four trials analyzed (Table 1). Overall response rate (ORR) of nab-paclitaxel arm ranged from 33% to 54%, whereas that of sb-paclitaxel and docetaxel arm ranged from 19% to 38%. Intention-to-treat analysis demonstrated higher ORR of nab-paclitaxel compared with sb-paclitaxel and docetaxel, but this difference was not statistically significant (risk ratio [RR] = 1.36, 95% confidence interval [CI] = 0.94–1.98, *P* = 0.11) (Figure 2A). However, considerable heterogeneity was observed across studies (*I^2^* = 83.3%, *P* < 0.001). Thus, we reported pooled RR from random-effects model.

We performed post hoc sensitivity analysis to evaluate influence of including studies from the same database. Including only one study from the same database for overall response showed no significant effect on pooled RR. Other potential sources of heterogeneity, including use of bevacizumab as combination drug, were examined by meta-regression analysis. However, we detected no significant factor (*P* = 0.12). Neither Begg's funnel plot nor Egger's test regarding response rate indicated existence of publication bias (Begg's test, *P* = 0.31; Egger's test, *P* = 0.11).

### OS and survival probability

Median OS was demonstrated in all four studies, with values ranging from 15.2 months to 33.8 months for nab-paclitaxel arm and from 13 months to 26.6 months for sb-paclitaxel and docetaxel arm. Pooled hazard ratio (HR) for OS in studies showed no significant differences between the two arms (HR = 1.06, 95% CI = 0.93–1.21, *P* = 0.38; *I^2^* = 20.9%) (Figure 2B).

The above studies published Kaplan–Meier curves of OS. Meta-analysis of one-year and two-year survival probability detected no significant differences between the two arms (one-year RR = 1.00, 95% CI = 0.83–1.21, *P* = 1.00; *I^2^* = 0%; two-year RR = 1.04, 95% CI = 0.90–1.21, *P* = 0.57; *I^2^* = 51.2%) (Figure 2C and 2D). Begg's and Egger's tests regarding survival confirmed absence of publication bias (Begg's test, OS *P* = 0.73, one-year *P* = 1.00, two-year *P* = 1.00; Egger's test, OS *P* = 0.68, one-year *P* = 0.58, two-year *P* = 0.59).

### Grade 3 to 4 toxicities

**Figure 3:**
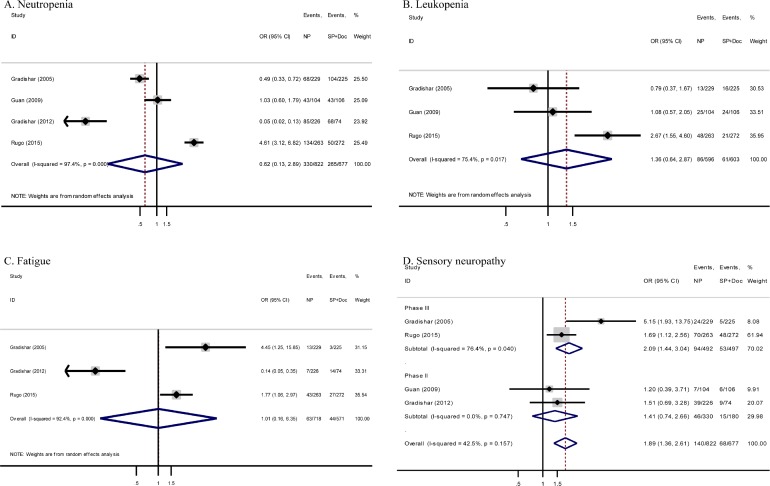
Forest plots of ORs for comparison between nab-paclitaxel and conventional taxanes in MBC patients stratified by different adverse effects (**A**) neutropenia, (**B**) leukopenia, (**C**) fatigue and (**D**) sensory neuropathy. NP, nab-paclitaxel; SP, sb-paclitaxel; Doc, docetaxel; CI, confidence interval; OR, odds ratio.

All trials provided toxicity profiles in a per-patient manner. One trial skipped obtaining complete data for fatigue [17], whereas another trial also failed to include information for leukopenia [19]. Risk of grade 3 to 4 neutropenia, fatigue, and leukopenia was almost comparable between the two arms (odds ratio [OR] = 0.62, 95% CI = 0.13–2.89, *P* = 0.54; OR = 1.01, 95% CI = 0.16–6.35, *P* = 0.99; and OR = 1.36, 95% CI = 0.64–2.87, *P* = 0.42, respectively) (Figure 3A, 3B, and 3C, respectively).

Meta-analysis of grade 3 to 4 hematological and non-hematological toxicities detected no significant differences between the two arms except the more prominent sensory neuropathy for nab-paclitaxel-based chemotherapy (16.9% vs. 10.0%, OR = 1.89, 95% CI = 1.36–2.61, *P* < 0.001; *I^2^* = 42.5%). As shown in Figure 3D, similar results were observed in meta-analysis of two randomized phase III trials (19.1% vs. 10.7%, OR = 2.09, 95% CI = 1.44–3.04, *P* < 0.001; *I^2^* = 76.4%). However, meta-analysis of two randomized phase II trials showed non-significant difference (13.9% vs. 8.3%, OR = 1.41, 95% CI = 0.74–2.66, *P* = 0.30; *I^2^* = 0%).

### Subgroup analysis

To explore possible reasons for any observed heterogeneity, we also conducted the following prespecified subgroup analyses: treatment arm (two groups: comparing nab-paclitaxel with sb-paclitaxel and comparing nab-paclitaxel with docetaxel), study phase (phase II vs. phase III), treatment line (first line vs. non-first line), and study location (Europe and America vs. East Asia). All subgroup results agree with outcomes except that ORR to nab-paclitaxel-based chemotherapy was significantly higher than to sb-paclitaxel and docetaxel-based chemotherapy in the subgroup of randomized phase II trials (RR = 1.50, 95% CI = 1.18–1.91, *P* = 0.001), non-first-line treatment (RR = 1.80, 95% CI = 1.42–2.29, *P* < 0.001), and East Asian population (RR = 1.84, 95% CI = 1.30–2.60, *P* = 0.001). Table 2 summarizes all other subgroup analyses.

**Table 2:** Subgroup analysis of the meta-analysis

Outcomes	Factor	Studies,*n*	Effect (95% CI)	*P*-value	Heterogeneity	Model used
Overall response	Treatment arms					
	NP + SP	3	1.41 (0.84–2.36)	*P* =0.19	*I^2^* = 88.8%, *P*< 0.001	R
	NP + Doc	1	1.23 (0.88–1.74)	*P* =0.23	N/A	R
	Study phase					
	Phase II	2	1.50 (1.18–1.91)	*P* =0.001	*I^2^* = 61.4%, *P* = 0.11	F
	Phase III	2	1.25 (0.63–2.46)	*P* =0.53	*I^2^* = 91.4%, *P* = 0.001	R
	Treatment line					
	1st line	2	0.99 (0.82–1.19)	*P* =0.89	*I^2^* = 58.2%, *P* = 0.12	F
	Non-1st line	2	1.80 (1.42–2.29)	*P* < 0.001	*I^2^* = 0%, *P* =0.89	F
	Study location					
	Europe and America	3	1.24 (0.81–1.87)	*P* =0.32	*I^2^* = 83.2%, *P* = 0.003	R
	East Asia	1	1.84 (1.30–2.60)	*P* =0.001	N/A	R
	Overall	4	1.36 (0.94–1.98)	*P* =0.11	*I^2^* = 83.3%, *P*< 0.001	R
Overall Survival	Treatment arms					
	NP + SP	3	1.02 (0.89–1.18)	*P* =0.76	*I^2^* = 2.1%, *P* =0.36	F
	NP + Doc	1	1.32 (0.93–1.87)	*P* =0.12	N/A	F
	Study phase					
	Phase II	2	1.17 (0.89–1.55)	*P* =0.27	*I^2^* = 19.4%, *P* = 0.27	F
	Phase III	2	1.03 (0.89–1.20)	*P* =0.69	*I^2^* = 48.3%, *P* = 0.16	F
	Treatment line					
	1st line	2	1.21 (1.00–1.48)	*P* =0.05	*I^2^* = 0%, *P* =0.57	F
	Non-1st line	2	0.94 (0.78–1.13)	*P* =0.52	*I^2^* = 0%, *P* =0.97	F
	Study location					
	Europe and America	3	1.07 (0.93–1.23)	*P* =0.33	*I^2^* = 43.6%, *P* = 0.17	F
	East Asia	1	0.95 (0.60–1.51)	*P* =0.83	N/A	F
	Overall	4	1.06 (0.93–1.21)	*P* =0.38	*I^2^* = 20.9%, *P* = 0.29	F
one-year survival	Treatment arms					
	NP + SP	3	1.00 (0.82–1.22)	*P* =0.97	*I^2^* = 0%, *P* =0.65	F
	NP + Doc	1	0.96 (0.49–1.88)	*P* =0.91	N/A	F
	Study phase					
	Phase II	2	1.05 (0.70–1.58)	*P* =0.80	*I^2^* = 0%, *P* =0.74	F
	Phase III	2	0.99 (0.80–1.22)	*P* =0.90	*I^2^* = 0%, *P* =0.41	F
	Treatment line					
	1st line	2	1.08 (0.79–1.47)	*P* =0.65	*I^2^* = 0%, *P* =0.71	F
	Non-1st line	2	0.96 (0.76–1.22)	*P* =0.73	*I^2^* = 0%, *P* =0.52	F
	Study location					
	Europe and America	3	0.98 (0.80–1.21)	*P* =0.87	*I^2^* = 0%, *P* =0.71	F
	East Asia	1	0.96 (0.76–1.22)	*P* =0.69	N/A	F
	Overall	4	1.00 (0.83–1.21)	*P* =1.00	*I^2^* = 0%, *P* =0.83	F
two-year survival	Treatment arms					
	NP + SP	2	1.02 (0.77–1.36)	*P* =0.87	*I^2^* = 69.3%, *P* = 0.07	R
	NP + Doc	1	1.23 (0.84–1.80)	*P* =0.29	N/A	F
	Study phase					
	Phase II	1	1.23 (0.84–1.80)	*P* =0.29	N/A	F
	Phase III	2	1.02 (0.77–1.36)	*P* =0.87	*I^2^* = 69.3%, *P* = 0.07	R
	Treatment line					
	1st line	2	1.20 (0.98–1.47)	*P* =0.07	*I^2^* = 0%, *P* =0.89	F
	Non-1st line	1	0.89 (0.72–1.10)	*P* =0.28	N/A	F
	Study location					
	Europe and America	3	1.04 (0.90–1.21)	*P* =0.57	*I^2^* = 51.2%, *P* = 0.13	F
	East Asia	0	N/A	N/A	N/A	N/A
	Overall	3	1.04 (0.90–1.21)	*P* =0.57	*I^2^* = 51.2%, *P* = 0.13	F

Abbreviations: NP, nab-paclitaxel; SP, sb-paclitaxel; Doc, docetaxel; CI, confidence interval; N/A, not applicable; R, random effects model; F, fixed effects model.

## DISCUSSION

As the first meta-analysis to compare nab-paclitaxel and conventional taxanes (sb-paclitaxel and docetaxel) in treatment of MBC, we failed to demonstrate ORR and survival advantages of nab-paclitaxel-based chemotherapy compared with sb-paclitaxel and docetaxel-based chemotherapy. Subsequently, we further probed results in subgroup analyses stratified by treatment arm, study phase, treatment line, and study location. We demonstrated that patients from randomized phase II trials, non-first-line treatment or East Asia yielded significantly higher overall response to nab-paclitaxel-based chemotherapy. Difficulty arose from interpretation of these results, especially across different clinical studies. Efficiency between nab-paclitaxel and conventional taxanes in other types of cancers remains controversial. Socinski et al. carried out a phase III trial including 1,052 untreated patients with stage IIIB to IV *non-small cell lung cancer* [22]. Nab-paclitaxel demonstrated significantly higher ORR than sb-paclitaxel in patients with squamous histology, but differences between progression-free survival (PFS) and OS were not statistically significant in the two arms. Palmieri et al. showed that two-year disease-specific survival and OS were significantly better in nab-paclitaxel-based induction chemotherapy compared with docetaxel-based induction chemotherapy in p16-positive advanced squamous cell carcinoma of the head and neck [23]. Given the lack of sufficient evidence for direct comparison, applying indirect comparison or network meta-analysis was considered for further investigation.

Considering that no statistical significance in survival was observed between nab-paclitaxel and conventional taxanes, efficacy may not be the only factor that may influence decision of physicians when selecting between the two types of treatments. Toxicity profiles and costs should also be considered for patients with MBC. Toxicity analyses indicated that equivalent tolerance was observed between the two chemotherapies with regard to grade 3 to 4 toxicities, except that sensory neuropathy was significantly more prominent in nab-paclitaxel-based chemotherapy. According to Dranitsaris et al. [24], when the median number of cycles delivered from clinical trials was applied, cost per course of nab-paclitaxel totaled $19,752 compared with $8,940 and $13,741 for sb-paclitaxel and docetaxel, respectively. Evidently, nab-paclitaxel treatment proves to be uneconomical because of severe sensory neuropathy, costs, and equivalent efficacy of other chemotherapies. However, in some circumstances, use of nab-paclitaxel is advised especially in patients with diabetes mellitus, as its administration does not require corticosteroids [25].

The latest phase III study, CALGB 40502/NCCTG N063H, recruited 799 patients, who were randomized to receive (i) paclitaxel (90 mg/m^2^), (ii) ixabepilone (16 mg/m^2^), or (iii) nab-paclitaxel (150 mg/m^2^), with or without bevacizumab (98% of patients received bevacizumab), as first-line treatment of MBC for 3 weeks in a 4-week period [20]. No significant differences were observed between nab-paclitaxel and standard paclitaxel arms in this trial. These results differed from findings reported in the phase III trial by Gradishar et al. [13] and may be due to different study populations (chemotherapy-naive vs. pretreated patients), addition of bevacizumab, and varying doses and schedules of taxanes (nab-paclitaxel and standard paclitaxel). Without direct evidence, earlier trials led to widespread use of more costly and higher-dose nab-paclitaxel in many clinical practices. Based on our analysis, conventional taxanes should remain the preferred microtubule inhibitors for treating patients with MBC, coinciding with results of Rugo et al. [20]. An earlier meta-analysis comparing sb-paclitaxel-based versus docetaxel-based regimens demonstrated that both regimens exhibited comparable efficacy for MBC patients in terms of OS, PFS, TTP, and ORR, but fewer grade 3 or 4 adverse events, including anemia, neutropenia, febrile neutropenia, thrombocytopenia, mucositis, diarrhea, and fatigue were observed in the sb-paclitaxel-based regimen [26]. Our meta-analysis results serve to remind clinicians when incorporating new agents, particularly those that are expensive and potentially toxic.

However, some limitations exist in our meta-analysis. For instance, analyses centered on extracted data and not original data. Specifically, an original data-based meta-analysis may produce more reliable estimation for association; therefore, investigators should carefully study our results, especially for positive association in subgroup analyses. Additionally, publication bias may exist, though none was observed based on stable results revealed in graphical and statistical methods. Considering that the major role of chemotherapy in patients with MBC is palliative, influence on patients’ quality of life (QOL) is also a decisive factor in assessing intrinsic value of this therapy. Nevertheless, none of the four trials conducted official QOL evaluations. Hence, further investigations will bear significance in assessing differences in QOL between the two regimens. Finally, the current meta-analysis is limited by insufficient quantity of randomized clinical trials, and more prospective trials are needed to confirm obtained results.

In conclusion, the current meta-analysis failed to demonstrate superiority of nab-paclitaxel to sb-paclitaxel and docetaxel in patients with MBC. The newer agent was associated with increased sensory neuropathy, equivalent survival, and possible increased overall response for some specific patients.

## MATERIALS AND METHODS

### Literature search

A systematic literature search was performed using PubMed/Medline, EmBase, Cochrane Library, and China National Knowledge Infrastructure, and October 20, 2016 was set as cut-off date. The search included the following terms: “albumin-bound paclitaxel,” “nab-paclitaxel,” “abraxane,” “ABI-007,” and “breast cancer.” No language restrictions were used. Reference lists of review articles included studies, and the Physician Data Query registry of clinical trials was carefully searched to identify additional potentially eligible studies. We reviewed each publication, and only the most recent or complete reports were included as duplicate publications were identified.

### Inclusion and exclusion criteria

Meta-analysis of evaluation for efficacy and toxicity of taxane-based regimens included potentially eligible randomized controlled trials comparing treatment arm containing nab-paclitaxel with those containing sb-paclitaxel or docetaxel in patients with stage IIIB–IV breast cancer. Trials were potentially eligible regardless of line of treatment. Studies published in abstract form only were excluded because of lack of efficacy and toxicity data. Assessment of eligibility criteria was performed independently by three investigators. Differences were resolved by consensus.

### Validity assessment

An open assessment of the trials was performed using the instrument reported by Jadad et al. [21].

### Data extraction

Data abstraction was conducted independently by two investigators (Y. Liu and G.X. Ye) according to the Preferred Reporting Items for Systematic Reviews and Meta-Analyses (PRISMA) [27], and any discrepancies between reviewers were resolved by consensus. Recorded data included study characteristics(first author, journal, year of publication), trial design characteristics (study design, treatment line, therapy regimen for each arm), study population (median age, number of randomly assigned patients in each arm, and percentage of patients in performance status 0–1), efficacy results (complete response and partial response for overall response, HRs, and 95% CIs for survival data), and adverse event results (type and number of adverse events in each treatment arm). Several trials did not provide an HR or CI for survival data. Therefore, two investigators independently analyzed Kaplan–Meier curves of trials to calculate these data using the method described by Parmar et al. [28]. Primary outcomes were ORR, OS, and survival probability. Secondary outcomes were specific toxicity data, such as neutropenia, sensory neuropathy, leukopenia, and fatigue.

### Statistical analysis

All statistical analyses were performed using Stata version 12.0 software (StataCorp, College Station, TX, USA). For survival variables such as OS, we used HR and corresponding 95% CI, which were presented as forest plots. For categorical variables, we used RR, OR, and corresponding 95% CI, which were presented as forest plots. Results were tested for heterogeneity and were considered significant at *P* < 0.05. Fixed-effects (weighted with Mantel–Haenszel) and random-effects models were considered for meta-analyses. The latter was calculated using the method of DerSimonian and Laird; this method considers both within-study and between-study variation [29]. For each meta-analysis, statistical heterogeneity among studies included in meta-analysis was first assessed using Cochrane's Q statistic, and inconsistency was quantified with *I^2^* statistic [30]. When *P* value was less than 0.1, assumption of homogeneity was deemed invalid; in this case, we reported summary estimates from random-effects models. Otherwise, fixed-effects model was reported. For a four-arm study [19], we combined arms that included nab-paclitaxel to create a single pair-wise comparison.

Meta-regression was performed to explain some heterogeneity, and subgroup analyses were conducted for potential confounding factors, which were selected by reviewing characteristics of included studies. All subgroup analyses followed the same meta-analysis procedure. Finally, potential publication bias was evaluated through Begg's funnel plots and Egger's test to examine relative symmetry of individual study estimates around overall estimate [31, 32]. A two-tailed *P* value of less than 0.05 was considered statistically significant. This article followed the Quality of Reporting of Meta-analyses statement, Cochrane Collaboration, and PRISMA guidelines for reporting meta-analyses [33, 34].
